# Hospitalisation for degenerative cervical myelopathy in England: insights from the National Health Service Hospital Episode Statistics 2012 to 2019

**DOI:** 10.1007/s00701-022-05219-5

**Published:** 2022-05-05

**Authors:** Edward Goacher, Richard Phillips, Oliver D. Mowforth, Stefan Yordanov, Erlick A. C. Pereira, Adrian Gardner, Nasir A. Quraishi, Antony H. Bateman, Andreas K. Demetriades, Marcel Ivanov, Alexandru Budu, Edward Dyson, Guy Wynne-Jones, Benjamin M. Davies, Mark R. N. Kotter, NIHR POLYFIX-DCM Consortia

**Affiliations:** 1 Department of Neurosurgery, Hull University Teaching Hospitals NHS Trust, Hull, UK; 2 Goffin Consultancy, Canterbury, UK; 3 Academic Neurosurgery Unit, Department of Clinical Neurosurgery, University of Cambridge, Cambridge, UK; 4 Department of Neurosurgery, St George’s, University of London, London, UK; 5 The Royal Orthopaedic Hospital NHS Foundation Trust, Birmingham, UK; 6 Centre for Spinal Studies & Surgery, Queen’s Medical Centre, Nottingham, UK; 7 Royal Derby Spinal Centre, University Hospitals of Derby and Burton NHS Foundation Trust, Derby, UK; 8 Department of Neurosurgery, New Royal Infirmary, Edinburgh, UK; 9 Edinburgh Spinal Surgery Outcome Studies Group, Edinburgh, UK; 10 Department of Neurosurgery, Sheffield Teaching Hospitals NHS Foundation Trust, Sheffield, UK; 11 Department of Neurosurgery, University Hospitals Birmingham, Birmingham, UK; 12 National Hospital of Neurology and Neurosurgery, London, UK; 13 Newcastle Upon Tyne Hospitals NHS Foundation Trust, Newcastle upon Tyne, UK; 14 Division of Neurosurgery, Department of Clinical Neurosciences, Addenbrooke’s Hospital, University of Cambridge, Cambridge, UK

**Keywords:** Cervical, Myelopathy, Spondylosis, Degeneration, Disability

## Abstract

**Purpose:**

Degenerative cervical myelopathy (DCM) is the most common cause of adult spinal cord dysfunction worldwide. However, the current incidence of DCM is poorly understood. The Hospital Episode Statistics (HES) database contains details of all secondary care admissions across NHS hospitals in England. This study aimed to use HES data to characterise surgical activity for DCM in England.

**Methods:**

The HES database was interrogated for all cases of DCM between 2012 and 2019. DCM cases were identified from 5 ICD-10 codes. Age-stratified values were collected for ‘Finished Consultant Episodes’ (FCEs), which correspond to a patient’s hospital admission under a lead clinician. Data was analysed to explore current annual activity and longitudinal change.

**Results:**

34,903 FCEs with one or more of the five ICD-10 codes were identified, of which 18,733 (53.6%) were of working age (18–64 years). Mean incidence of DCM was 7.44 per 100,000 (SD ± 0.32). Overall incidence of DCM rose from 6.94 per 100,000 in 2012–2013 to 7.54 per 100,000 in 2018–2019. The highest incidence was seen in 2016–2017 (7.94 per 100,000). The median male number of FCEs per year (2919, IQR: 228) was consistently higher than the median female number of FCEs per year (2216, IQR: 326). The rates of both emergency admissions and planned admissions are rising.

**Conclusions:**

The incidence of hospitalisation for DCM in England is rising. Health care policymakers and providers must recognise the increasing burden of DCM and act to address both early diagnoses and access to treatment in future service provision plans.

## Introduction

Degenerative cervical myelopathy (DCM) is the most common cause of adult spinal cord dysfunction worldwide, estimated to affect 2% of the adult population [6, 10–12, 29]. DCM is caused when degenerative, arthritic and/or congenital processes stress and injure the spinal cord [14, 24]. This can lead to a range of disabilities, including imbalance and difficulty walking, loss of manual dexterity, sensory loss, bowel or bladder dysfunction, pain and, in extreme circumstances, paralysis [7, 12].

The current incidence of DCM is poorly understood but estimated to be around 4 per 100,000 population per year globally [28]. Most figures are based on operative incidence as surgical decompression is the mainstay of treatment, recommended for moderate, severe or progressive DCM [6, 10]. Whilst this is likely to be a major underestimate of overall incidence, given not all patients proceed to surgery, and DCM often goes undiagnosed [3, 19, 30, 32], the operative incidence has not previously been reported for the UK. Reporting this will add value in not only characterising the diagnostic gap, but also in projecting future surgical demand, which is particularly relevant for a condition positively associated with increasing age [9].

Similarly, increased knowledge of the true incidence of DCM will help predict the future economic impacts of the condition. A survey by Myelopathy.org (Charity No. 1178673) of its DCM community found all patients harbour disabilities despite treatment with 36% unable to work and 42% dependent on others for day to day care [5, 27]. Based on cases diagnosed today (7.4/100,000) the cost to UK society has been calculated as £685 m per year, including an average lifetime loss of earnings for those of working age of £0.5 m [4].

The Hospital Episode Statistics (HES) database contains details of all secondary care admissions, accident and emergency attendances and outpatient appointments across National Health Service (NHS) hospitals in England [23]. It is a ‘big data’ source from which data can be retrieved and analysed. The role for HES data has already been demonstrated in work analysing outcomes and costs associated with lumbar spinal surgery [33, 34].

This study aimed to use HES data to characterise DCM surgical activity in England, including current annual activity and longitudinal change.

## Methods

To establish the prevalence of DCM in England, we analysed the HES (NHS Digital) database from 2012 to 2019. This period reflects baseline practice, before the implications of the COVID-19 pandemic. The HES database compiles a record of every ‘episode’ during an admission across NHS England. More than one episode may be associated with an individual admission. HES data helps facilitate payment to NHS care providers, resource allocation and future resource planning across NHS England. NHS Digital is the national provider of data and IT systems for health and social care in NHS England [15].

Wishlist the HES data is made up of several datasets, detailing all admissions, accident and emergency attendances and outpatient appointments at NHS hospitals in England, only the Hospital Admitted Patient Care Activity dataset, provides data stratified by both diagnostic code and age. This dataset is available at: https://digital.nhs.uk/data-and-information/publications/statistical/hospital-admitted-patient-care-activity#about-this-publication [22]. Within the dataset, age-stratified values are provided for ‘Finished Consultant Episodes’ (FCEs), which correspond to a patient’s hospital admission under a lead consultant. Data was collected and compiled by one author (RP), as part of a wider report for Myelopathy.org, seeking to develop a burden of illness model in DCM [20, 25].

DCM cases were identified from 5 ICD-10 codes: M47.1 (Other spondylosis with myelopathy), M50.0 (Cervical disc disorder with myelopathy), M99.3 Osseous stenosis of neural canal), M99.4 (Connective tissue stenosis of neural canal) and M99.5 (Intervertebral disc stenosis of neural canal). These codes were author selected based on their ICD-10 definition being most closely aligned to the definition of DCM.

Graphs were generated from the data using IBM SPSS version 26.0 (IMB Corp., Armonk, NY).

## Results

In total, 34,903 FCEs diagnosed with one or more of the five ICD-10 codes were identified from the HES data analysed between 2012 and 2019.

### Incidence of DCM

**Fig. 1:**
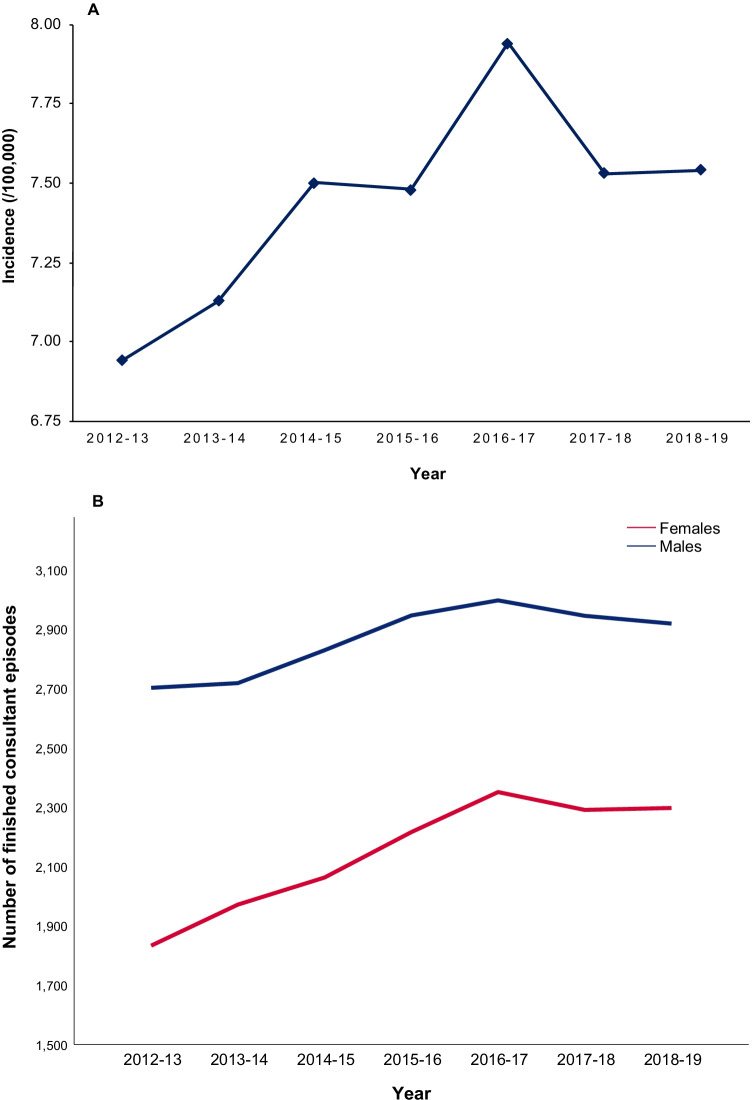
**A** Incidence of degenerative cervical myelopathy by year. **B** Number of finished consultant episodes by sex

The mean incidence of DCM was 7.44 (SD ± 0.32). Overall incidence of DCM rose from 6.94 per 100,000 in 2012–2013 to 7.54 per 100,000 in 2018–2019 (Table 1). Highest incidence was seen in 2016–2017 (7.94 per 100,000). With the exception of 2016–2017 to 2017–2018, incidence of DCM rose year on year (Fig. 1).

**Table 1:** Tabulated display of prevalence. Finished consultant episodes included the following ICD-10 codes: M47.1, M50.0, M99.3, M99.4 and M99.5

Year	Finished consultant episodes	Admissions	Incidence (/100,000)	Male:female proportion	Emergency:planned admission proportion
2012–2013	4535	3711	6.94	60%	19%
2013–2014	4689	3841	7.13	58%	20%
2014–2015	4892	4076	7.50	58%	17%
2015–2016	5162	4097	7.48	57%	19%
2016–2017	5348	4387	7.94	56%	17%
2017–2018	5236	4187	7.53	56%	20%
2018–2019	5216	4218	7.54	56%	20%

### Age

Overall, the mean age across the 34,903 FCEs was 62.1 years. Of the 34,903 FCEs, 0.1% (n = 54) were < 18 years old. 46.2% (n = 16,116) were aged > 65 years. The remaining 18,733 (53.6%) were of working age, between 18 and 64 years. Of the 18,733 aged 18–64, mean age was 51.3 years.

### Sex

The number of male FCEs for DCM was consistently greater than female episodes across the 7-year period examined (Fig. 1). Median male number of FCEs per year was 2919 (IQR = 228). The median female number of FCEs per year was 2216 (IQR = 326). However, the male to female (M: F) ratio does appear to be decreasing over time. In 2012–2013, M: F 60% of FCEs were male (2702/4535), by 2018–2019 this had steadily decreased to 56% (2919/5216).

### Emergency vs. planned admissions

**Fig. 2:**
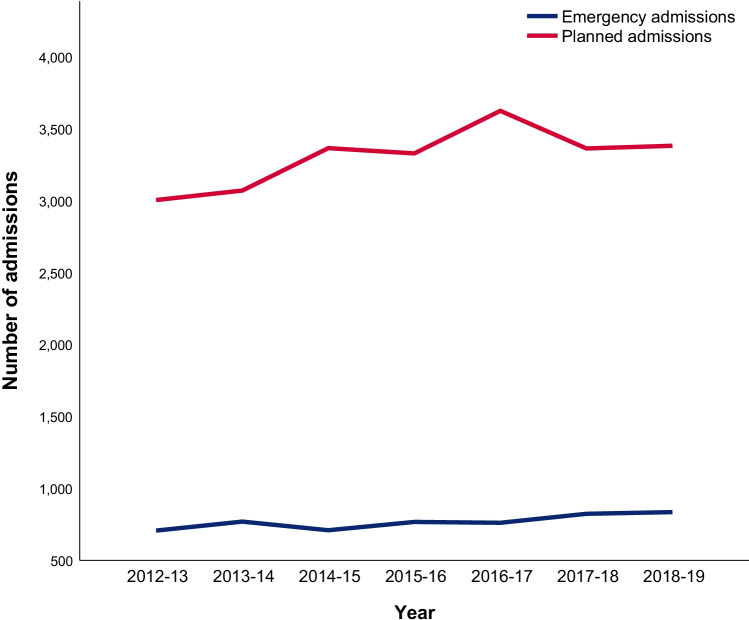
Number of emergency and planned admissions for DCM

The number of patients admitted as an emergency was consistently fewer than the number of planned admissions. The mean proportion of patients admitted as an emergency was 18.9% (SD ± 1.1%). Numbers for both emergency and planned admissions are rising (Fig. 2). In 2012–2013, 706 patients were admitted as an emergency and 3005 were planned admissions. By 2018–2019, these numbers had risen to 835 and 3383, respectively. On analysis of emergency admissions as a percentage of total admissions, there has been minimal change between 2012–2013 and 2018–2019 (19% vs. 20%, respectively, range: 17–20%).

### Waiting times

The mean waiting time for specialist review following referral from primary care for DCM between 2012 and 2019 was 73 days. Mean waiting time has been moderately stable throughout the 7 years between 50 and 90 days.

### Length of stay

Mean length of stay (LoS) was 5 days in all years except 2016–2017, when it rose to 7 days. In comparison, median LoS varied between 1 and 3 days, with the highest LoS again seen in 2016–2017. When examined by ICD-10 diagnosis, M47.1 (Other spondylosis with myelopathy), displayed consistently the highest mean LoS between 2012 and 2019. This was closely followed by diagnosis M50.0 (Cervical disc disorder with myelopathy). The lowest mean LoS was seen in the diagnoses M99.3 (Osseous stenosis of neural canal) and M99.4 (Connective tissue stenosis of neural canal).

## Discussion

This is the first study to utilise HES data to examine DCM activity within England. DCM was more common amongst men than women, and hospitalisation more likely through planned than emergency admission. DCM hospitalisation has risen since 2012, although more recently has plateaued. Further, the gender gap may also be closing. Importantly, observed activity falls far below recent estimates of DCM prevalence [29].

From the open access, population level data in HES, it is difficult to establish the exact nature of each hospital episode. However, the mainstay of treatment for DCM is surgery [7, 17] and broader experience, including regional audits, has demonstrated that the management of most patients within the NHS under a primary diagnostic code of DCM, is related to surgery [13, 14]. Our assumption therefore, is this data is mostly related to surgical activity, and this would accord with national figures from other nations [24]. These studies also demonstrate the higher prevalence of DCM amongst men than women [35].

This study examined 34,903 FCEs across 7 years. During this time, the overall incidence of DCM rose from 6.94 to 7.54 per 100,000 in England. Whilst one might assume this relates to an ageing population for a condition associated with age [7, 24], it is noteworthy that the cohort was mostly of working age (18–64), and the incidence more recently stable. Another assumption why the incidence is rising within this dataset could be the change in international guidelines for surgery and change in surgical attitude with expanding patients’ groups currently being offered treatment for DCM. A recent retrospective cohort study of patients at a single tertiary hospital found that prior to publication of the guidelines, only half of DCM patients who met the criteria for surgical decompression received surgery in routine practice. (12) These numbers also remain far smaller than recent best estimates of DCM prevalence (2.7%) (3). Based on an average time lived with disease of 20 years (UK life expectancy 81.2 minus average age of admissions 62.2), a 2.7% prevalence would equate to an expected incidence of 135/100,000 — 20-fold greater than observed. Whilst this will incorporate patients who do not require surgery, it remains a significant diagnostic gap.

Taken together these inconsistencies further support the notion that DCM is widely underdiagnosed. Patients who obtain a diagnosis of DCM will face significant delays and often have been misdiagnosed initially [13, 14, 27]. Addressing this delay is a major priority for DCM as the aim of surgery is principally to halt disease and symptom progression. Whilst most patients will experience some improvement following surgery, a longer time period with symptoms before the intervention leads to poorer outcomes [7]. The ratio of emergency to planned admissions was therefore noteworthy, including a small rise in the percentage of emergency admissions from 19 to 20% during the 7-years analysed. Unfortunately, the data granularity is not sufficient enough to assess if these emergency admissions were associated with previous FCEs or longer waiting times.

Prior analysis of UK referral pathways has shown that even from the point of referral from primary care, access to treatment will take over one year, with patients navigating an inconsistent network of professionals and investigations [14]. This is a widespread issue for neurological disease, leading to the release of NICE Clinical Guidance 127; Suspected neurological conditions: recognition and referral [21]. Unfortunately, DCM was not considered within this framework [21]. This data in this paper supports the need to address how individual’s access care, particularly as experience from the USA has demonstrated that patients presenting acutely and requiring emergency admission are more likely to require more complex surgery, with an increase LoS, greater post-operative rehabilitation needs and poorer outcomes [1, 2, 8, 16, 31]. Whilst unreported in the UK, this aligns with the experience of the UK authors.

This study had multiple limitations. Firstly, only HES data from 2012 and onwards was included in this study due to prior inconsistencies in data reporting. Whilst this ensured that only higher quality, consistent data was included, it truncated the time frame for which to examine activity trends. Secondly, interrogation of such ‘big data’ sources can be associated with a reduced level of data granularity and variability in standards between coders and hospitals which may lead to coding error, omitted data and duplications. A higher level of granularity would have allowed a more detailed analysis of emergency admissions and the impact of patient demographics on waiting times and length of stay. However, for the aims of this study — characterising surgical activity for DCM across England — the HES database provides adequate information to address the primary aim. Similarly, the cost implications of the increasing incidence of hospitalisation for DCM was not assessed, therefore commenting on its economic impact is beyond the scope of this study — future studies may wish to examine this. Furthermore, without a specific ICD code, DCM was queried using a number of defined surrogates felt most faithful to the definition of DCM. These have been used elsewhere [18, 26], albeit outside of England, but the exact coding practice for English hospitals is currently uncertain. It should be noted that such ICD-10 codes are not procedure specific and in practice, may not always be associated with optimal (surgical) management.

## Conclusion

Interrogation of ‘big data’ sources such as the HES database provides useful and substantial data for clinicians and health care providers to better comprehend and characterise the increasing demands of DCM on health care resources. This data has reinforced the challenges around access to care, with a high proportion of emergency admissions and long waiting times. Furthermore, it highlights the large gap between estimated and actual diagnosis, with a potential health inequality amongst the elderly which requires further evaluation. In short, it is imperative that health care policymakers and providers recognise the burden of DCM and act to address both early diagnosis and timely access to treatment in future service provision plans.

## Declarations

### Disclaimer

This report is independent research arising from a Clinician Scientist Award, CS-2015–15-023, supported by the National Institute for Health Research. The views expressed in this publication are those of the authors and not necessarily those of the NHS, the National Institute for Health Research or the Department of Health and Social Care.
